# Zymographic detection and clinical correlations of MMP-2 and MMP-9 in breast cancer sera

**DOI:** 10.1038/sj.bjc.6601725

**Published:** 2004-03-16

**Authors:** G La Rocca, I Pucci-Minafra, A Marrazzo, P Taormina, S Minafra

**Affiliations:** 1 Dipartimento di Oncologia Sperimentale e Applicazioni Cliniche, Università di Palermo, Viale delle Scienze, 90128 Palermo, Italy; 2 Centro di Oncobiologia Sperimentale (COBS), Università di Palermo, Viale delle Scienze, 90128 Palermo, Italy; 3 Casa di Cura di Alta Specialità, La Maddalena, Via S. Lorenzo, 90100 Palermo, Italy

**Keywords:** matrix metalloproteinases, gelatin zymography, breast cancer, clinic correlations

## Abstract

Matrix metalloproteinases, in particular the gelatinases MMP-2 and MMP-9, have received great attention in recent years as putative tumour markers for clinical applications. The main reason for the observed interest is their easy detection in body fluids. Moreover, recent evidence has shown multiple functions of MMPs, rather than simply degrading ECM, which include the mobilisation of growth factors and processing of surface molecules. Several authors have reported increased levels of MMPs in a number of cancers, but clinical correlations in breast cancer are still fragmentary. Thus, the aim of the present research was to investigate the activity levels of circulating gelatinases in the sera of breast cancer patients by means of zymographic analysis, and correlate data with clinicopathological parameters. In all, 80 patients and 22 healthy volunteers were involved in this study. Sera were obtained prior to surgery. The clinical variables were: grading of tumours, tumour size, lymph node involvement, tumour staging, oestrogen and progesterone receptor levels (76 out of 80 cases), and c-erbB-2 levels (46 cases). The densitometric measures of MMP-2 and MMP-9 activity levels indicated that the average values of both gelatinase activities were significantly higher in breast cancers than in control sera (*P*<0.0001). In addition, our analysis showed for the first time that elevated activity levels of both gelatinases correlated only with c-erbB-2 overexpression (*P*=0.0273 for MMP-2 and *P*=0.0075 for MMP-9). An inverse correlation was observed with regard to oestrogen receptor expression (*P*=0.0075 for MMP-2 and *P*=0.0273 for MMP-9). Moreover, a borderline inverse correlation was observed between the activity levels of both enzymes and nuclear grade (*P*=0.0511 for MMP-2 and *P*=0.0794 for MMP-9). In conclusion, the present data suggest that serum measures of MMP's activity may have diagnostic value for discriminating subgroups of breast cancer patients and support the hypothesis that *ERBB2* amplification and/or overexpression enhance signalling pathways that may lead to increased production of gelatinases in c-erbB-2 positive breast cancers with higher metastatic potentialities.

Tumours arise from multiple genetic alterations, which affect primarily cell proliferation. A crucial difference between benign and malignant tumours is that the first remain encapsulated for undefined periods of time and do not form metastases, while the second acquire the ability to invade the underlying basal lamina and its adjacent stroma, which in normal tissues do not show passageways for cells of epithelial origin.

A critical event, during progression of malignant carcinomas, is the invasive growth of neoplastic cells into the host tissues: this involves the onset of a number of complex interactions occurring at the tumour–host interface, including an extensive remodelling of the extracellular matrix (ECM). Degradation of the ECM requires the concerted action of a number of extracellular enzymes.

Several enzyme families are known to be involved in extracellular proteolysis. These include serine proteases (e.g. Blasi and Carmeliet, 2002; Diamandis and Yousef, 2002), matrix metalloproteinases (MMPs; reviewed by Somerville *et al*, 2003), and disintegrin-metalloproteinase (ADAMs: *A Disintegrin And Metalloproteinase Domain*; reviewed by O'Shea *et al*, 2003).

The MMP family is one of the most studied, since many members of the group have been recognised as involved in cancer invasion and metastasis. Recently, MMPs have been the object of renewed interest due to the additional roles attributed to their functions, for example, growth factor mobilisation and the processing of surface molecules (cf. Somerville *et al*, 2003). At present, there are 23 human MMP genes, which are structurally similar to each other, indicating that they evolved by duplication of a common ancestral gene followed by divergent evolution (Moura-da-Silva *et al*, 1996). Based on substrate specificity and domain organisation, the MMPs have been tentatively divided into four main groups: interstitial collagenases, gelatinases, stromelysins and membrane-type MMPs. Most MMPs are secreted as proenzymes and are organised into distinct structural domains, with some differences in domain composition and number (Nagase and Woessner, 1999).

Gelatinase A (MMP-2) and gelatinase B (MMP-9) differ from other MMPs in that they have three tandem fibronectin type II repeats within the amino terminus of the catalytic module that mediates gelatin binding (Murphy *et al*, 1994). Moreover, these two enzymes interact with physiological inhibitors (respectively, TIMP-2 and TIMP-1) even if they are in the proenzymatic form (Overall *et al*, 1999). Traditionally, MMP-2 and MMP-9 have been correlated with the invasive stage of carcinomas, because of their ability to degrade type IV collagen, a major constituent of basement membranes (Stetler-Stevenson, 1990). More recent evidence suggests that MMP-2 and MMP-9 may also be involved in breast cancer initiation and growth through complex interactions with the main oncogenes and tumour-suppressor genes involved in the early stage of tumorigenesis (Duffy *et al*, 2000; Leeman *et al*, 2003, for reviews). For example, transfection of MCF-10A breast cancer cells with either c-erbB-2 or c-ras resulted in increased expression of MMP-2 (Giunciuglio *et al*, 1995), whereas transfection of MCF-7 cells with the *ets* gene PEA-3 led to increased production of MMP-9 (Kaya *et al*, 1996). Due to the key role of MMPs in tumorigenesis, several authors have proposed MMP-2 and or MMP-9 as useful prognostic markers (i.e. Duffy, 1996). Recent work on breast cancer patients has suggested that MMP-2 negativity may be linked with a favourable prognosis in node-negative breast carcinoma (Hirvonen *et al*, 2003) and that high activity levels of plasma MMP-9 in breast cancer patients are associated with a worst overall survival rate (Ranuncolo *et al*, 2003). Thus, the present research was conducted with the following objectives: (1) to support the diagnostic value of MMP-2 and MMP-9 in breast cancer using a highly sensitive zymographic method; (2) to determine the possible association of activity levels of serum forms of MMP-2 and MMP-9 with the current clinical parameters; and (3) to perform the first univariate analysis including c-erbB-2, a candidate marker of tumour aggressiveness, at present in course of validation.

In the present study, we examined 80 patients with breast cancer and no detectable metastases. In all, 22 sera samples from healthy donors were used as control specimens. To assess possible correlations between the serum levels of MMP-2 and MMP-9 and the stage of the disease, the following clinical parameters were utilised: tumour size, lymph node involvement, tumour stage, histological grading, oestrogen receptor (ER) levels (76 out of 80 cases), progesterone receptor (PR) levels (76 cases), c-erbB-2 levels (46 cases). The statistical analyses have shown a significant increase of activity levels of both MMP-2 and MMP-9 in the sera of breast cancer patients compared with control sera. The correlation analyses indicated a significant association between high activity levels of both enzymes and c-erbB-2 overexpression.

## MATERIALS AND METHODS

### Sample collection

A total of 80 patients (of which one Tis, 52 T1, 25 T2, two T3) diagnosed with breast carcinoma but without clinically apparent metastases were involved in this study. Sera were obtained prior to surgery, according to the ethical standards, with informed consent of patients at the Maddalena Hospital. Native serum was prepared using plastic tubes without coagulation accelerators, to prevent the release of gelatinases during platelet activation (Fernandez-Patron *et al*, 1999). Tubes were centrifuged at 1600 **g** for 10 min, 30 min after blood collection. For each sample, determination of protein concentration was performed using the method of Bradford (1976). Sera were aliquoted and stored at −80°C until used. Each aliquot was used only once in order to prevent enzyme activation due to freeze–thawing processes. For all patients, the histological diagnosis and the stage of cancer were established by assessment on paraffin sections at diagnostic laboratory of the Maddalena Hospital. Evaluation of oestrogen receptor (ER) and progesterone receptor (PR) was performed by immunohistochemistry using monoclonal antibodies from Dako Corporation (USA). c-erbB-2 expression levels were evaluated using the HercepTest assay (semiquantitative immunocytochemical assay), with scoring of 0 or 1+ for normal levels of expression and 2+ or 3+ for overexpression (DakoCytomation, DK). Control sera (*n*=22) were taken from healthy volunteers.

### Gelatin zymography

Gelatin zymography was performed for both healthy control and cancer patients sera as follows: gels (SDS–PAGE, 7.5%) were copolymerised with gelatin (0.1%) (Sigma-Aldrich). For each sample, 28 *μ*g of total serum protein was loaded. Electrophoresis was carried out using the minigel slab apparatus Mini Protean 3 (Biorad) at a constant voltage of 150 V, until the dye reached the bottom of the gel. Following electrophoresis, gels were washed in renaturation buffer (2.5% Triton X-100 in 50 mM Tris–HCl (pH 7.5)) for 1 h in an orbital shaker. Then the zymograms were incubated for 18 h at 37°C in incubation buffer (0.15 M NaCl, 10 mM CaCl_2_, 0.02% NaN_3_ in 50 mM Tris–HCl (pH 7.5)). Gels were then stained with Coomassie blue and destained with 7% methanol and 5% acetic acid. Areas of enzymatic activity appeared as clear bands over the dark background.

### Quantification of enzymatic activity

Following zymography, the degree of gelatin digestion was quantified using a Sharp JX-330 scanner equipped with a transparency option interfaced to an IBM PC. Gels were scanned using Image-master software, version 1.2 for DOS (Pharmacia Biotech), in a grey scale mode at 169 *μ*m pixel size and 1250–1650 (*X*–*Y*) pixel count, using the autodensity feature on a scale ranging from 0 (clear) to 255 (opaque). The image was digitally inverted, so that the integration of bands was reported as positive values. The pixel density was determined after background subtraction and used to calculate the integrated density of a selected band. Values of integrated density were reported in volume units of pixel intensity per mm^2^. The integrated density of each band is reported as the mean of three different measurements of the same gel for each sample run in triplicate.

### Statistical analysis

For each group of subjects, data derived from zymographic quantification of activity of MMP-2 and MMP-9 were plotted using MS Excel software. Statistical analyses were performed using both MS Excel and Graph Pad Prism 4 (demo) software (for correlation analysis). Fitness of data to normal distribution was assessed using the method of Kolmogorov and Smirnov. This normality test quantifies the discrepancy between the examined distributions of data and an ideal Gaussian distribution; the test returns a *P*-value which is considered acceptable for values greater than 0.05. For all the distributions examined (i.e. activity levels of MMP-2 and MMP-9 in control and affected subjects), the test confirmed their fitness with a normal distribution. In order to estimate the significance of differences between cancer patients and control subjects, unpaired Student's *T*-test (with Welch's correction) was applied. Correlation of MMP-2 and MMP-9 activity levels with clinicopathological variables for breast cancer patients was performed using the Pearson correlation test. In all cases, data were considered significant for values of *P*<0.05.

### Ion exchange chromatography (batch procedure)

DEAE sephacryl has been used to prefractionate sera from breast cancer patients in order to reach a final purification of gelatinases. Serum samples (1 ml) were dialysed against equilibration buffer (50 mM Tris/HCl, 0.8% Brij-35 (pH 8)). The resin was rinsed thoroughly in the same buffer. Then, the sample and resin were placed in a tube and mixed for 1 h at 4°C with gentle inversion. At the end, resin was briefly centrifuged in order to recover the supernatant (unbound fraction) and successive washes with equilibration buffer were performed in order to remove all unbound proteins. All the steps were monitored by measuring *A*_280_ using an Eppendorff biophotometer. Two intermediate washes with 50 mM Tris/HCl (pH 8) were performed to eliminate detergent from the system. The resin was mixed at 4°C with the elution buffer (150 mM NaCl and 50 mM Tris/HCl (pH 8)) for 15 min, and then centrifuged to obtain the supernatant (bound fraction). Elution passages were repeated until *A*_280_ values near to zero, in order to elute all bound proteins. Finally, both bound and unbound fractions were dialysed against MilliQ water at 4°C. Zymography showed the presence of gelatinases only in bound fractions.

### Affinity chromatography (gelatin sepharose with batch procedure)

Lyophilised bound protein fractions from DEAE chromatography were solubilised in equilibration buffer (50 mM Tris/HCl, 1 M NaCl pH 7.5). After resuspension, the sample was added to resin and mixed at 4°C for 3 h. The resin was briefly centrifuged and then washed with equilibration buffer to eliminate all unbound proteins. Elution of gelatinases was accomplished by using a 10% DMSO solution; fractions were then dialysed against MilliQ water for lyophilisation and Western blotting analysis.

### Western blotting

After electrophoresis, proteins were electrotransferred on nitrocellulose membranes (Amersham) using the following transfer buffer: 25 mM Tris, 190 mM glycine and 20% methanol. Protein transfer was performed at 50 V for 1 h at 4°C. Following transfer, the nitrocellulose membrane was stained using Ponceau Red (0.2% Ponceau Red, 3% TCA) and destained with milliQ water. Blocking of the membrane was achieved using 5% nonfat milk in TTBS (pH 7.6). The primary antibody was incubated overnight at 4°C in 1% nonfat milk in TTBS. The antibodies used were purchased from Calbiochem (Ab-3 for anti-MMP-2 and Ab-7 for anti-MMP-9). Following incubation, the filter was washed six times for 5 min to remove the unbound antibody. Then the membrane was incubated with horseradish-peroxidase-conjugated antibody diluted 1 : 3000 in 1% nonfat milk in TTBS for 1 h at room temperature. After secondary antibody incubation, the membrane was washed six times for 5 min with TTBS. The filter was then incubated with luminescent substrate (Supersignal West Pico Pierce) and exposed to an appropriate film (Amersham).

### Inhibition assays

Zymograms of both breast cancer and healthy subjects sera were incubated with the described incubation buffer, added with chemical inhibitors of MMPs (EDTA 20 mM or 1,10 phenantroline 10 mM). Following incubation, at 37°C for 18 h, gels were stained with Coomassie blue.

## RESULTS

### Gelatin zymography

**Figure 1:**
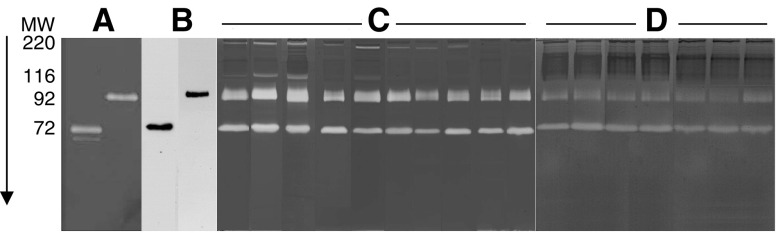
Panel of representative gelatin zymograms of sera from breast cancer patients and healthy subjects. (**A**) Standards of purified pro-MMP-2 (72 kDa) and pro-MMP-9 (92 kDa); (**B**) Western blotting of pro-MMP-2 and pro-MMP-9 from breast cancer sera; (**C**) sera of breast cancer patients; (**D**) sera of healthy subjects.

Sera from all subjects, both affected and healthy ones, were subjected to gelatin zymography to determine the relative levels of activity attributable to pro-MMP-2 and pro-MMP-9. Figure 1

shows a panel of zymograms from healthy and pathologic subjects, randomly selected from the collection of our samples, run in parallel with purified standards of pro-MMP-2 and pro-MMP-9, and a representative Western blot of purified gelatinases revealed with anti-MMP-2 and anti-MMP-9 monoclonal antibodies. The majority of breast cancer sera show stronger intensity of gelatinolytic bands for both the proenzymatic forms of MMP-2 (72 kDa) and MMP-9 (92 kDa) when compared with control sera. Moreover, most breast cancer sera display two lytic bands of 200 and 116 kDa, respectively, absent in the majority of the control sera assayed. These two lytic forms are comparable to those previously detected in colon carcinoma sera (Pucci-Minafra *et al*, 2001) and identified as MMP-9 dimers (the 220 kDa) and as MMP-9/TIMP1 complex (the 116 kDa).

### Identification of gelatinolytic activities

The nature of lytic bands observed in zymograms was further confirmed by both inhibition and immunodetection assays. Selective inhibitors of MMPs excluded the presence of gelatinolytic activities due to serine or cysteine proteinases. Incubation of the zymograms with 20 mM EDTA or 1,10 phenanthroline inhibited all the gelatinolytic activities of serum samples from both patients and volunteers (data not shown).

The immunologic detection of both enzymes was performed by Western blotting with monoclonal antibodies against MMP-2 and MMP-9, on purified gelatinases (described in Materials and methods) from serum samples. The antibodies recognised the proforms of both enzymes in purified sera of breast cancer patients (cf. Figure 1B), but not other forms corresponding to the higher molecular weight lytic bands.

### Quantitative analysis of gelatinolytic activity

**Figure 2:**
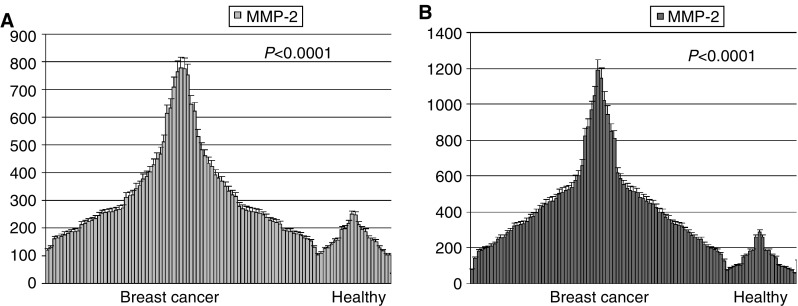
Diagrams of distribution of pro-MMP-2 (**A**) and pro-MMP-9 (**B**) activity in sera of the two groups. Values are indicated as OD for 1 *μ*g of total serum proteins. Values of density are calculated as mean of triplicate experiments with indication of the standard error.

Following gelatin zymography, gels containing samples run in triplicate were subjected to densitometric analysis to quantify the relative activity levels of the two gelatinases. Densitometric data in OD were then normalised for 1 *μ*g of total serum proteins and plotted in graph using MS Excel software. The Student's *T*-test (with Welch's correction) was applied in order to compare the differences between the two groups. As shown in Figure 2

, the average activity values of both pro-MMP-2 and -9 are significantly higher in breast cancer sera *vs* the control sera (*P*<0.0001). In detail, the basic statistical features describing differences in enzyme activity levels between breast cancer patients (BC) and control group (C) were the following:

MMP-9: mean=412.3^BC^ *vs* 141.7^C^; media*n*=347^BC^ *vs* 124.5^C^; s.d.=239^BC^ *vs* 65.59^C^

MMP-2: mea*n*=320.1^BC^ *vs* 160.7^C^; media*n*=260^BC^ *vs* 154.5^C^, s.d.=168.3^BC^ *vs* 45.82^C^.

### Correlation of serum gelatinase A and B levels with clinical parameters

In order to assess the possible correlations between levels of gelatinase activity and tumour stage, statistical analysis was performed using Pearson correlation. The variables considered for this analysis were: grading of tumours, tumour size, lymph node involvement, tumour staging (following AJCC guidelines) and oestrogen and progesterone receptor levels. These are some of the prognostic factors that currently direct adjuvant therapy administration (Goldhirsch *et al*, 2001). In addition, we analysed the correlation of activity of both gelatinases with c-erbB-2 receptor level (for 46 out of 80 samples, 57.5%). As shown in Table 1

**Table 1:** Table of the clinicopathological variables assessed for primary tumours

**Factors**	**No of patients**	**%**
*Tumour size*		
Tis	1	1.25
T1	52	65
T2	25	31.25
T3	2	2.5

*Lymph node involvement*		
N0	47	58.75
N1	31	38.75
N2	2	2.5

*Staging*		
0	1	1.25
I	38	47.5
IIa	20	25
IIb	19	23.75
IIIa	2	2.5

*Grading*		
G1	6	7.5
G1–G2	4	5
G2	44	55
G2–G3	11	13.75
G3	15	18.75

*Oestrogen receptor level (76 cases)*		
Negative (<10%)	40	52.6
Positive (>10%)	36	47.4

*Progesterone receptor level (76 cases)*		
Negative (<10%)	41	54
Positive (>10%)	35	46

*cErbB-2 levels (46 cases)*		
0	33	71.7
1+	4	8.7
2+	2	4.3
3+	7	15.3

, the cancer patients displayed a variable clinical profile in good agreement with current literature: in particular, node involvement was demonstrated for about 40% of studied cases, and c-erbB-2 overexpression was limited to approx. 20% patients.

**Figure 3:**
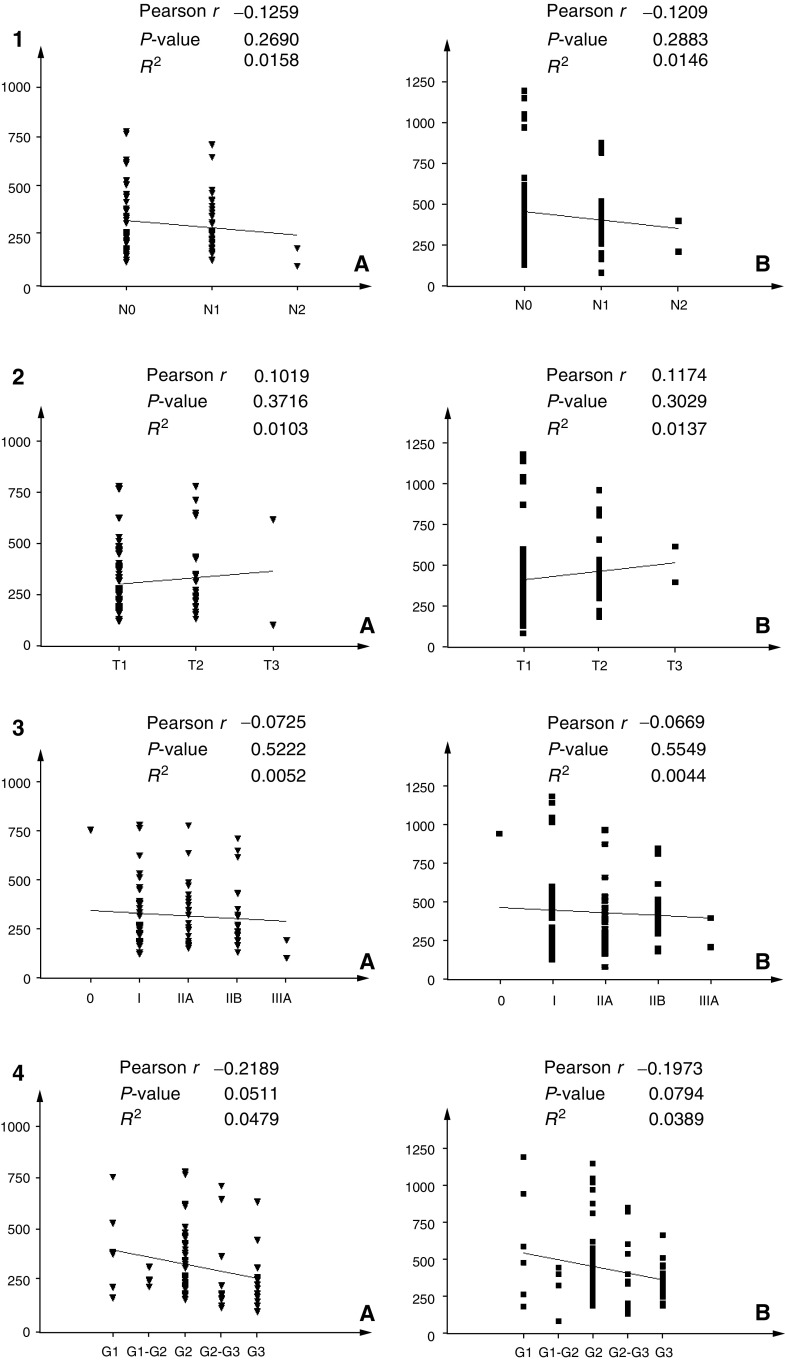
Correlation analysis between clinicopathological variables and progelatinase activity levels: For all variables, graphs marked with (**A**) indicate correlation with pro-MMP-2, while (**B**) indicates pro-MMP-9. (1) Correlation with lymph node involvement; (2) correlation with tumour size; (3) correlation with tumour staging; (4) correlation with tumour grading.

As shown in Figures 3

and 4

**Figure 4:**
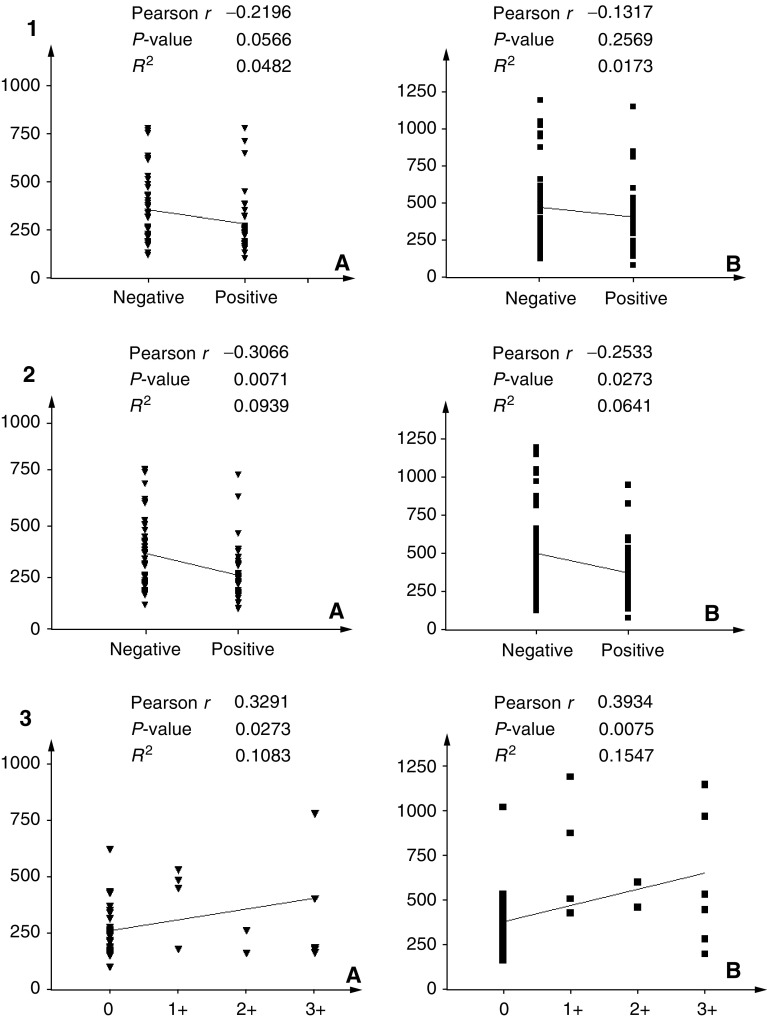
Correlation analysis between the receptorial state of primary tumour and serum progelatinase activity levels: (**A**) indicates pro-MMP-2, while (**B**) indicates pro-MMP-9. (1) Correlation with progesterone receptor positivity; (2) correlation with oestrogen receptor positivity; (3) correlation with c-erbB-2 expression levels.

, the activity of MMP-2 and MMP-9 in the sera of breast cancer patients did not correlate with tumour size, stage, node involvement and progesterone receptor levels. Instead, we found a negative correlation with ER expression (*P*=0.007 for MMP-2 and 0.027 for MMP-9). By contrast, a positive correlation was observed between gelatinolytic levels and c-erbB-2 overexpression (*P*=0.027 for MMP-2 and 0.007 for MMP-9). Moreover, a borderline significant negative correlation was found with nuclear grade (*P*=0.0511 for MMP-2 and 0.0794 for MMP-9).

## DISCUSSION

At present, the strongest predictors of breast cancer metastasis are lymph node involvement and histological grading. These parameters are not enough selective to discriminate the putative subgroup of patients within the same clinical category. In fact, it is well documented that breast cancer patients with the same stage of disease can have markedly different outcome and therapy responses (van't Veer *et al*, 2002). Therefore, searching for new molecular markers is an open area of interest. In particular, the matrix metalloproteases have since long attracted the interest of investigators, due to their possible use as molecular markers and therapeutic targets (cf. e.g. King *et al*, 2003). The majority of MMPs are secreted as latent proenzyme forms and are subjected to regulated activation at the cell–matrix boundary (Nagase and Woessner, 1999). Their proenzyme forms are also secreted in body fluids where they can be easily detected.

Several authors, including our group (Pucci-Minafra *et al*, 2001), have measured the serum or plasma levels of MMP-2 and MMP-9 in oncologic patients (e.g. Garbisa *et al*, 1992; Zucker *et al*, 1993; Hayasaka *et al*, 1996; Garbett *et al*, 1999; Oberg *et al*, 1999), in some cases reaching different conclusions. One reason could be the different pre-analytical sampling process (reviewed by Jung *et al*, 2001, 2002). Indeed, plasma preparation requires the addition of anticoagulants such as EDTA or heparin, while serum is often obtained by addition of clot accelerator. All these additives may interfere with measurements of gelatinase activity. In particular, EDTA is a strong inhibitor of MMPs and heparin is known to bind to some MMPs (Keller *et al*, 1986; Wallon and Overall, 1997). On the other hand, serum preparation with clot accelerators can induce platelets to release MMP-9 (Jung *et al*, 2001); this can be avoided by preparing native serum in the absence of clot accelerators, since quiescent platelets release negligible amounts of gelatinases (Fernandez-Patron *et al*, 1999).

On this ground, our major aim was to study the gelatinolytic levels of serum forms of MMP-2 and MMP-9 by zymographic assays, and correlate data with current clinicopathological parameters. These zymographic tests have some advantages over immunologic assays, due to lower costs, more rapid time of execution and the possibility of detecting simultaneously multiple forms of the same enzyme.

Our qualitative analysis of the zymograms showed that the majority of the 80 sera samples from breast cancer patients displayed sharp bands of lysis corresponding to the proenzyme forms of MMP-2 and MMP-9, respectively, in contrast to the 22 control samples which showed less pronounced lytic bands. In addition, the sera samples from breast cancer patients exhibited two other lytic activities of larger molecular size, similar to those previously identified as pro-MMP-9 dimers (200 kDa) and as a complex formed between pro-MMP-9 and its physiological inhibitor TIMP-1 (116 kDa) in colon cancer sera (Pucci-Minafra *et al*, 2001). These forms were absent or very faint in the majority of normal sera. Activated forms of both enzymes were never detected in any sera.

The densitometric analyses indicated that the average levels of activity of circulating MMP-2 and MMP-9 in breast cancer patients were significantly higher than control sera (*P*<0.0001), and suggest that serum measures of MMP-2 and MMP-9 activity may have diagnostic value for discriminating subgroups of breast cancer patients.

In order to relate our data with disease features, we applied statistical analyses correlating activity levels of both enzymes with clinical parameters. Univariate analysis revealed no correlation between both gelatinases and tumour size, staging, lymph node involvement and PR status, while a borderline correlation was observed with nuclear grading. Conversely, our data showed for the first time an inverse correlation between ER positivity (generally considered as a favourable prognostic factor) and elevated levels of gelatinase activity. More interestingly, high levels of gelatinases are significantly related to c-erbB-2 overexpression. c-erbB-2 overexpression has been described as an independent predictor of survival in breast cancer (Konecny *et al*, 2001), and its amplification has been reported for 20–25% of breast cancer patients (Dickson and Lippman, 1995) associated with more aggressive clinicopathologic features (Sahin, 2000). In addition, recent clinical investigations (Berney *et al*, 1998; Allgayer *et al*, 2000) demonstrated an association between ERBB2 overexpression and tumour-associated proteolysis in gastric and colon cancer, suggesting a direct role for ERBB2 in invasion and metastasis through upregulation of proteolytic enzymes. However, the association of ERBB2 expression with the expression of MMP-2 and MMP-9 has not previously been reported in breast cancer. The present data support the hypothesis that ERBB2 amplification and/or overexpression enhance signalling pathways that may lead to increased production of gelatinases in c-erbB-2-positive breast cancers with higher metastatic potentialities. With regard to biological significance, it appears reasonable to think that the suggested network between c-erbB-2 pathways and MMPs overexpression by *in vitro* models (Giunciuglio *et al*, 1995) may be operative also during tumour progression.
